# Neurology

**Published:** 1900-05

**Authors:** William C. Krauss

**Affiliations:** Buffalo, N. Y.


### Progress in Medical Science.

### NEUROLOGY.

CONDUCTED BY WILLIAM C. KRAUSS, M. D., Buffalo, N. Y.

### THYROID EXTRACT IN INSANITY.

DRS. MABON, superintendent, and Babcock, assistant physician (*American Journal of Insanity*, October, 1899), St. Lawrence State Hospital, make a careful review of the results obtained in the treatment of 1,032 collected cases of insanity by thyroid extract and submit the following propositions:

1. The dose of the extract depends entirely on the individual case. In some cases twenty-five grains, three times a day will be necessary to bring about a circulatory or temperature-reaction; while in others the same result may be had with the use of five grains, *t.i.d.* Each case must be a law unto itself.

2. It is essential that the patient should be placed in bed to obtain the best results, and he should be continued there during the entire treatment and for a week following its discontinuance.

3. The treatment should be continued for at least thirty days.

4. We should not be discouraged by failure in the first administration, but should resort to two, three, or more trials, if necessary.

5. The most gratifying results in thyroid treatment are to be obtained in cases of acute mania and melancholia with prolonged attacks, puerperal and climacteric insanities, stuporous states and primary dementia, particularly where these forms of mental alienation do not respond to the usual methods of treatment.

6. A high temperature-reaction is not essential, as we found the average maximum temperature in the recovered cases among men was 99.6 degrees.

7. Physical improvement is the outcome in most cases whether mental improvement takes place or not.

8. The proportion of individuals who recover under thyroid treatment and then relapse is less than the proportion that relapse after recovery from other methods of treatment. In our own series of cases, only one patient who recovered has relapsed.

---

### RENAL DISEASE AND MENTAL DERANGEMENT.

W. L. Worcester, of Danvers (*American Journal of Insanity*, October, 1899,) Insane Hospital, Danvers, Mass., makes a searching inquiry into the relations between renal disease and mental derangement, presenting statistics from various sources confirming the conclusion that chronic renal disease follows essentially the same law in the sane and the insane, increasing in both with advancing years.

In considering the significance of renal disease, coincident with mental derangement, the following possibilities must, it seems to him, be taken into account :

1. The relation may be a mere coincidence. If it is true, as would seem probable from the foregoing statistics, that the frequency of disorders of the kidneys is not in very marked excess in the insane over that in the general population, it would seem probable that such is the fact in a great proportion of cases.

2. The renal disease may be the immediate cause of the mental symptoms. It may, I think, be laid down as a general proposition, that any poison which is capable of causing coma may also produce slighter degrees of mental disturbance. That the so-called uremic condition may be the cause of active mental derangement is, I think, unquestionable. Probably such cases are more commonly seen in private than in hospital practice, as they are apt to be accompanied by such evident physical signs of renal disease as to make their nature plain, but they are occasionally found in hospitals for the insane. The mental symptoms, in the cases I have recognised, have consisted, as might perhaps be anticipated, in mental confusion, with a depressed emotional tone, and at times, a tendency to violence in the effort to escape from misunderstood surroundings or imaginary dangers.

3. The renal disease and the mental disturbance may both result from a common cause. In the few cases of "acute delirium" in which I have had an opportunity to examine the urine, it has been, I think without exception, heavily charged with albumin, and has contained large quantities of casts. The pathology of these cases is obscure, and is very possibly not the same in all the cases presenting the symptom-complex of high temperature, great motor restlessness, utter mental confusion and rapid exhaustion; but whether the disorder is primarily toxic or neuropathic, it seems to me unlikely that the starting point is in the kidneys.

The conclusions provisionally arrived at may be summarised as follows:

Renal disease, in some degree, is very common among the insane, but it is by no means certain that it is very much more common among them than in the population at large at corresponding ages.

Cases in which insanity is due simply to disease of the kidneys are rather infrequent in hospitals for the insane. Taking the term "Bright's disease" in its broadest significance, however, it is probably one of the most common causes, if not the most common, of mental derangement.

